# Identification of potential key protein interaction networks of BK virus nephropathy in patients receiving kidney transplantation

**DOI:** 10.1038/s41598-018-23492-2

**Published:** 2018-03-22

**Authors:** Linpei Jia, Wenjing Fu, Rufu Jia, Leiyun Wu, Xiaoxia Li, Qiang Jia, Hongliang Zhang

**Affiliations:** 1 Department of Nephrology, Xuanwu Hospital of Capital Medical University, Changchun Street 45#, 100053 Beijing, China; 2 Central Hospital of Cangzhou, Xinhua Middle Street 201#, 061001 Cangzhou, Hebei Province China; 3 Department of Life Sciences, the National Natural Science Foundation of China, Shuangqing Road 83#, 100085 Beijing, China

## Abstract

We aim to identify the key protein interaction networks and implicated pathways of BK virus nephropathy (BKVN) via bioinformatic methods. The microarray data *GSE75693* of 30 patients with stable kidney transplantation and 15 with BKVN were downloaded and analyzed by using the *limma* package to identify differentially expressed genes (DEGs). Then the gene ontology (GO) functional enrichment analysis and the Kyoto Encyclopedia of Genes and Genomes (KEGG) pathway analysis were done to investigate the molecular function (MF), biological process (BP), cellular components (CC) and pathways of DEGs. Finally, protein-protein interactions (PPIs) were constructed, and the hub proteins were  identified. As a result, 249 up-regulated genes and 253 down-regulated genes of BKVN patients were selected based on criteria of *P* > 0.01 and fold change >2.0. GO and KEGG showed that DEGs were mainly located in nucleus and cytosol, and were implicated in the immune responses. In the PPI analysis, 26 up-regulated and 8 down-regulated proteins composed the pivotal interaction network. CXCL10, EGF and STAT1 were identified as hub proteins in BKVN. In conclusion, CXCL10, EGF and STAT1 may induce kidney injuries by promoting inflammation and prohibiting reparation of tissue damage in BKVN.

## Introduction

The BK virus (BKV) is a double-stranded DNA virus, belonging to the *Polyomaviridae* family^1^. Antibodies against BKV were detectable in more than 90% of children by age 10, indicating an asymptomatic infection of BKV during the early childhood^2^. Once the primary infection occurs, BKV persists latently in the renal epithelium^3^. BKV can be reactivated after kidney transplantation and leads to BK virus nephropathy (BKVN), which is characterized by interstitial fibrosis and cellular infiltration^4,5^. BKVN is one of the main causes of graft dysfunction and morbidity in renal-transplant recipients^6,7^. The cause of increasing incidence of BKVN is still unknown^7^. At present, excessive immunotherapy, e.g. tacrolimus and mycophenalate mofetil, might be the primary risk factors of BKVN^8–10^.

The common therapy for BKVN is the reduction of immunosuppression, which may result in severe acute graft rejection^1^. Leflunomide combined with everolimus or intravenous immunoglobulin may be safe rescue therapies of BKVN^6,11^. However, these therapies have not been proved in preclinical experiments or large randomized controlled studies. Thus, it is important to look into the mechanism of the disease and find out the key point of BKVN for new therapeutic targets.

A few studies have explored the pathogenesis of BKVN, including the innate and the adaptive immune systems. Some researchers showed that the increasing number of dendritic cells could inhibit the immune evasion of BKV, increase magnitude of virus-specific CD8^+^ T cells and enhance the natural killer cells-mediated cytotoxicity in immune responses to BKV^12–14^. Some immune factors also participate in the pathogenesis of BKVN, such as interleukin-6 (IL-6), IL-8/CXCL8, RANTES/CCL5, MCP-1/CCL2, and IP-10/CXCL10^15^. However, previous studies simply demonstrated that expression levels of these cell factors were changed (Supplementary Table S1), but failed to display the detailed mechanisms, e.g. what are the biological functions of these factors, how they interact with each other and which cell factor plays a key role in the interaction network.

Bioinformatics is a kind of tool to collect, classify and analyze biological datasets such as the gene expression microarray dataset^16,17^. Gene expression analysis by bioinformatic methods has been widely used in genomics and biomedical research, providing insights into the molecular events underlying human biology and disease^18^. Data mining of the available microarray  datasets could help scientists to narrow down the research scope and to carry out targeted experiments.

In this study, we analyzed the public array data by bioinformatics methods to find out the important gene network of BKVN. Differentially expressed genes (DEGs) were first identified between stable and BKVN renal-transplantation recipients. Then protein-protein interactions (PPIs) were further analyzed. Finally, we attempted to identify the key genes and to obtain better insights into the pathogenesis of BKVN.

## Results

### Five hundred and twenty-four DEGs were selected

**Figure 1:**
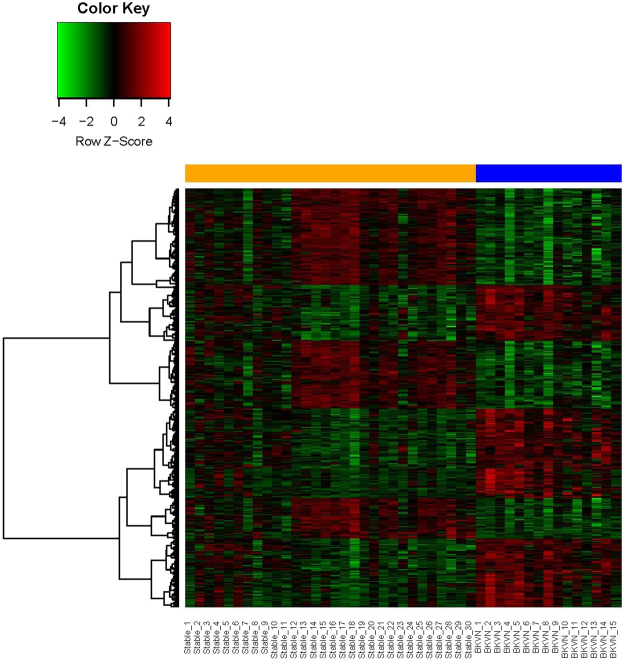
Heatmap of differentially expressed genes. Each column represents a sample, and each row represents a gene. Above the heatmap, yellow bar represents samples of stable renal allograft patients, and blue bar represents samples of BKVN patients. In the heatmap, green means down-regulation, while red means up-regulation. Color black means no difference expressed in this gene between BKVN and stable allograft patients.

Microarray data of BKVN and stable kidney transplantation patients were compared by the *limma* package by the linear model, the contrast model and the DEG selection. A total of 502 DEGs were selected according to the criteria of *P* < 0.01 and fold change >2.0, which include 249 up-regulated genes and 253 down-regulated genes (Supplementary Dataset S1). The hierarchical cluster analysis was done to show the distribution of DEGs (Fig. 1). Above the heatmap, the yellow bar represents samples of stable renal allograft patients, and the blue bar represents samples of BKVN patients. In the heatmap, each column represents a tissue sample, and each row represents a single gene. The gradual color from green to red means the changing degree from down-regulation to up-regulation. Color black means no difference expressed in this gene between patients with BKVN and with stable allografts.

### DEGs of BKVN are mainly enriched in the immune response

**Figure 2:**
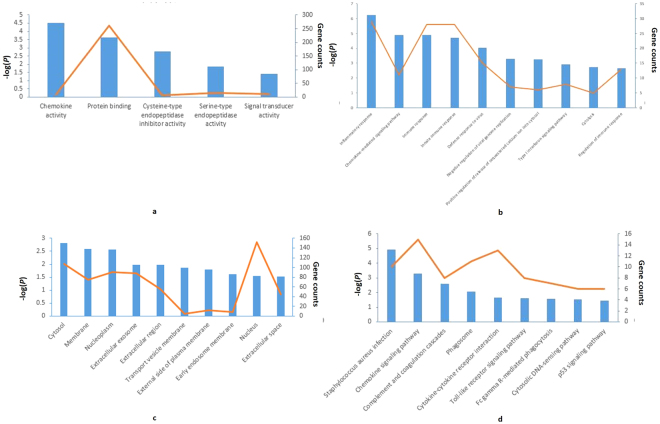
Results of gene ontology and Kyoto Encyclopedia of Genes and Genomes (KEGG) analyses. Blue bar chart represents the value of −log (*P*), whilst the orange line chart represents the gene count of each category. (**a**) Results of molecular function analysis. (**b**) Results of biological process analysis. (**c**) Results of cellular component analysis. (**d**) Results of KEGG pathway analysis.

To investigate biological functions of DEGs, we further analyzed DEGs in DAVID with criteria of *P* < 0.05 and the count ≥5, including MF, BP, CC and KEGG pathway. In MF ontology, DEGs mainly enriched in 5 categories about protein interactions (Supplementary Dataset S2, Fig. 2a), such as the protein binding (262 genes), the serine-type endopeptidase activity (14 genes) and the signal transducer activity (11 genes). In BP ontology, the majority enriched categories are the negative regulation of transcription from RNA polymerase II promoter (31 genes), the inflammatory response (29 genes), the innate immune response (28 genes) and the immune response (28 genes), which are focused on the immune process. Since a total of 39 categories are involved in the BP analysis, only top 10 categories in the gene count were shown in Supplementary Dataset S3 and Fig. 2b. CC ontology displays the distribution of DEGs in cells. According to results of CC analysis, proteins of DEGs are mostly located in the nucleus (152 genes) and the cytosol (107 genes). Other important CC categories are the extracellular exosome, the nucleoplasm, the membrane and so  forth (Supplementary Dataset S4, Fig. 2c). Furthermore, 17 dysfunctional pathways in BKVN are found out by KEGG pathway analysis (Supplementary Dataset S5, Fig. 2d). Important pathways are the chemokine signaling pathway (15 genes) and the phagosome (11 genes).

According to results of the enrichment, DEGs between BKVN and stable renal allograft patients centrally locates in nucleus and cytosol, and probably participate in the protein interactions process in the immune response.

### Fourteen significant genes constructed co-citation network in literature mining

**Figure 3:**
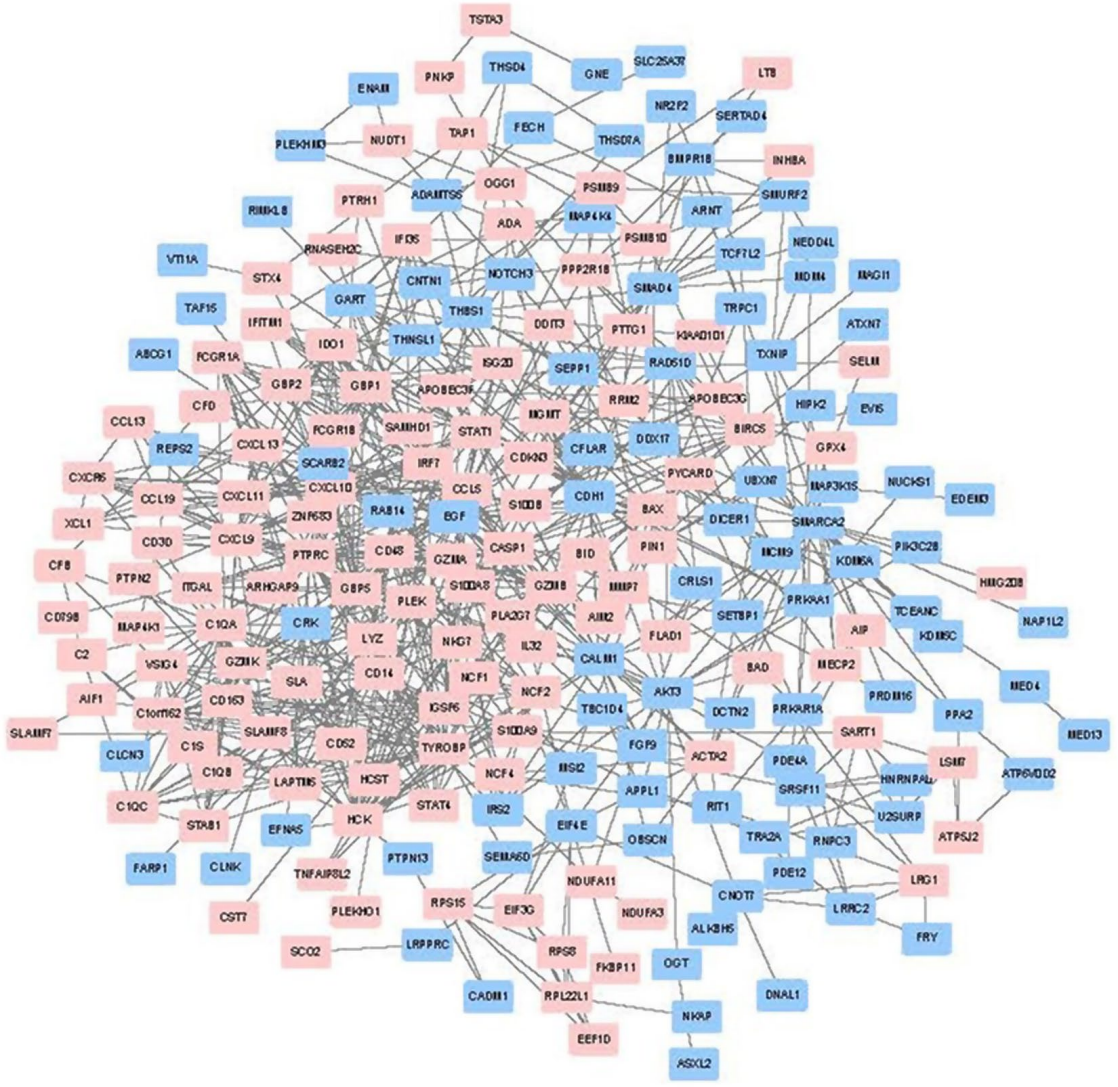
The protein-protein interaction network was constructed with deferentially expressed genes. Red represents up-regulated genes in BKVN patients compared with stable allograft patients, and blue represents down-regulated genes.

**Figure 4:**
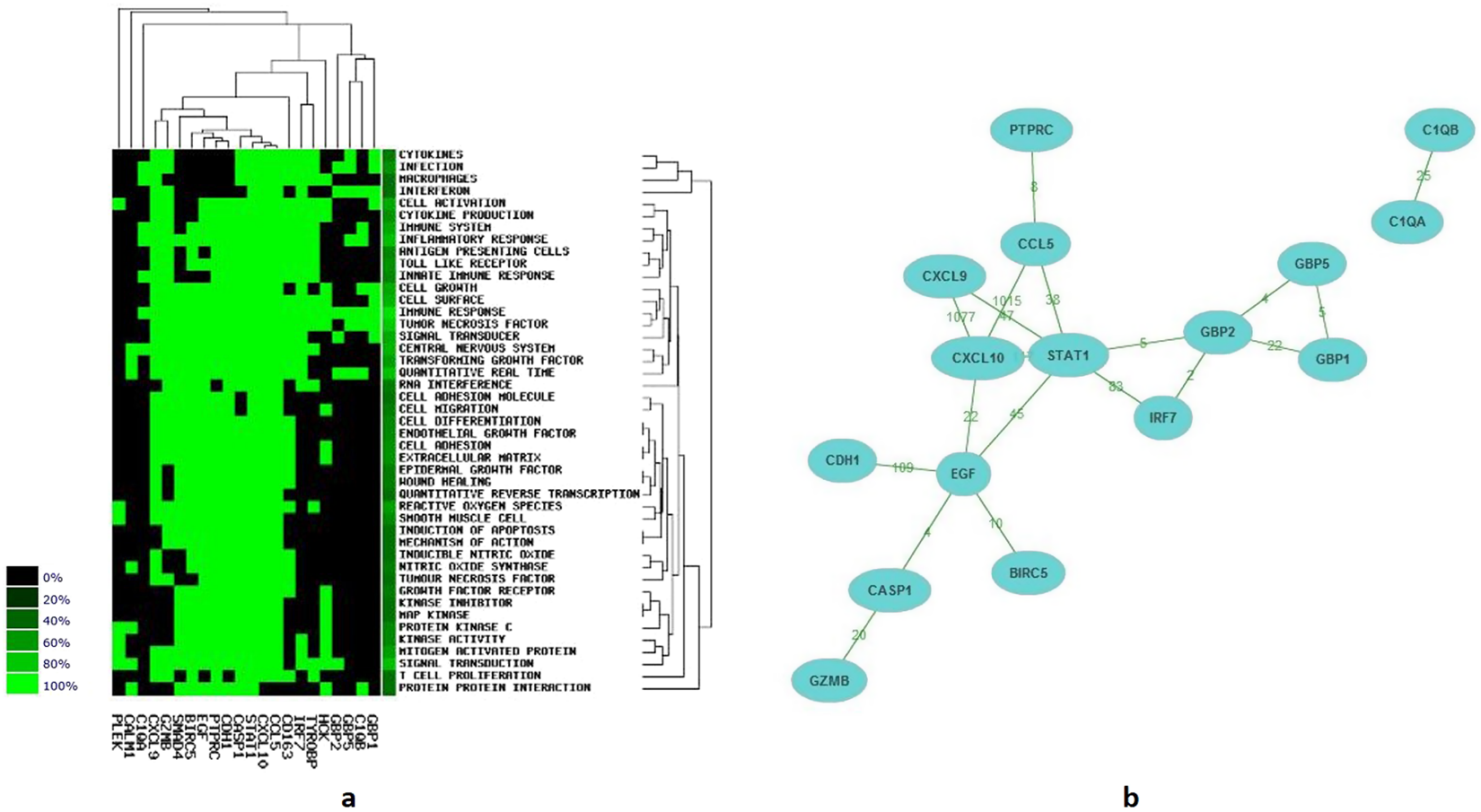
Literature mining results of proteins with degree ≥10. (**a**) Clustering analysis of biological functions of 22 genes in previous studies. In the heatmap, color black means that the biological function of the gene has not been reported yet. While color light green means that the gene has the biological function according to previous studies. Hot genes mainly clustered in the immune response, the tumor necrosis factor and the signal transduction. (**b**) Co-citation network of hot genes in protein-protein interaction. In the co-citation network, 14 genes closely interacted, while *C1QA* only interacted with *C1QB*. The numbers noted on the line indicated number of studies co-cited.

At first, 502 DEGs were uploaded onto the STRING website. Then 219 DEGs with score >0.4 (median confidence) were selected to construct the PPIs (Fig. 3). In PPIs, genes closely associated with others were identified with the degree ≥10^19^, including *EGF*, *TYROBP*, *PTPRC*, *STAT1*, *CXCL10*, *HCK*, *CCL5*, *IRF7*, *CXCL9*, *GBP5*, *PLEK*, *CD163*, *SMAD4*, *CASP1*, *CDH1*, *GBP2*, *GBP1*, *BIRC5*, *GZMB*, *C1QB*, *CALM1* and *C1QA*. In the clustering analysis, main biological keywords of hot genes reported in literature were immune responses, tumor necrosis factor and signaling transductions (Fig. 4a). Among 22 significant genes, 16 genes constructed a co-citation network according to previous studies. However, in the network, *C1QA* and *C1QB* only interacted with each other, but not with the other 14 genes (Fig. 4b, Table 1).

**Table 1:** Hub genes identified by literature mining.

Gene	Co-genes (n)	Co-citations (n)	Total (n)
*STAT1*	6	280	6044
*EGF*	5	188	19511
*CXCL10*	4	1956	5290
*GBP2*	4	30	80
*CCL5*	3	1047	7812
*GBP5*	2	7	22
*CXCL9*	2	1093	1808
*GBP1*	2	25	176
*IRF7*	2	84	910
*CASP1*	2	24	4748
*GZMB*	1	20	3237
*C1QB*	1	25	89
*PTPRC*	1	8	9773
*C1QA*	1	25	94
*CDH1*	1	109	14418
*BIRC5*	1	10	6375

### CXCL10, EGF and STAT1 are significant genes in BKVN

**Figure 5:**
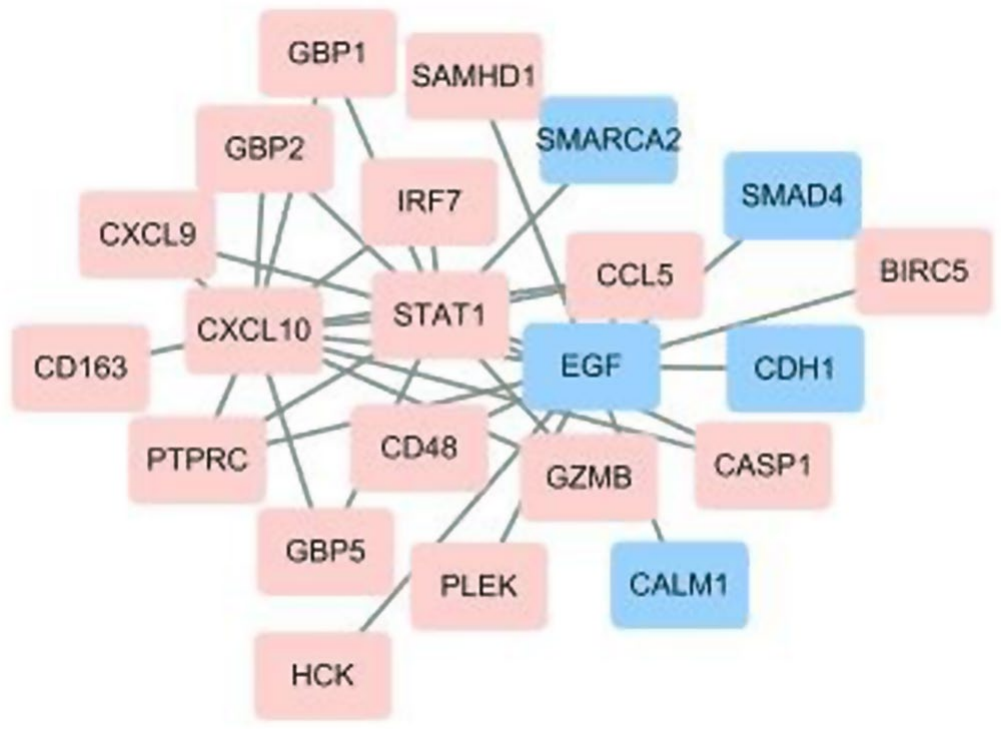
The protein-protein interaction (PPI) network of important proteins. Red represents up-regulated genes, while blue represents down-regulated genes. The PPI network consists of 17 up-regulated proteins and 5 down-regulated proteins. CXCL10, EGF and STAT1 are identified as hub proteins.

In the CytoNCA analysis, every DEG was scored according to degree centrality, betweenness centrality and subgraph centrality respectively (Table 2). Based on the results of CytoNCA analysis, CXCL10, EGF and STAT1 were chosen as hub proteins. The network of CXCL10, EGF, STAT1 and proteins directly associated with hub proteins are described in Fig. 5, including 17 up-regulated and 5 down-regulated proteins.

**Table 2:** Top 5 genes evaluated by degree centrality, betweenness centrality and subgraph centrality in the protein-protein interaction network.

Protein	Degree centrality	Protein	Betweenness centrality	Protein	Subgraph centrality
EGF	22.46	EGF	7640	CXCL10	1329.08
TYROP	18.27	PLEK	5007	STAT1	1089.18
PTPRC	18.22	SMARCA2	4470	CXCL9	1023.23
STAT1	16.10	CDH1	3679	CCL5	1015.45
CXCL10	15.98	EIF4E	3653	IRF7	1005.53

## Discussion

In this study, we aimed at finding out the key protein interaction networks in BKVN after kidney transplantation. By comparing the array datasets between BKVN and non-rejection transplantation patients, 267 up-regulated and 257 down-regulated DEGs were identified. Then the GO and KEGG analyses show the important role of innate immune system in BKVN. Finally, PPIs were constructed by 219 DEGs and 22 key proteins were selected, including CXCL10, EGF and STAT1.

By the GO annotation in DAVID, we further analyzed biological functions of DEGs, which helped us to infer the pathogenesis of BKVN. First, in the MF ontology, the enriched ontologies focused on the alteration of protein activities, including the protein binding, protein homodimerization activity, serine-type endopeptidase activity and the receptor activity. Both protein homodimerization and serine-type endopeptidase could activate or inhibit signaling pathways by changing structures of important proteins, such as receptors^20^, and further affect cellular processes, including inflammation, cell death and development^21,22^. In the BP ontology, the majority of DEGs were enriched in immune process, such as innate immune response, the inflammatory response and the immune response. It has been reported that BKVN is associated with the innate and specific immune system^1^. BKV may lead to nephropathy via cell lysis, stimulation of the immune system and induction of inflammation^23^. As one of the DEGs and a ligand of CXCL10, CXCR3 is expressed on T cells, dendritic cells and natural killer cells, and can stimulate the migration and activation of these immune cells in immune responses against BKV^24–26^. In the CC ontology, proteins of DEGs are mostly located in the nucleus and the cytosol. When BKV infects host cells, the virus enters into the host nucleus and lies episomally^1^. Once reactivated, BKV regulates the transcription of host cells. The other important CC category is the extracellular exosome, which contains all type of biomolecules, including proteins, lipids and so forth^27^. A number of pathogen-derived components, even RNAs, have been found in exosomes after viral infection^28^. Exosomes were involved in virus transmission in the infection process. However, little is known about the functions of exosomes in BKVN. Exosomes might offer new insights into the inhibition strategies against BKV reactivation. Taken together, DEGs may affect the structural changes of proteins in nucleus, cytosols and exosomes to participate the immune response in BKVN.

Both the literature mining and the CytoNCA analysis revealed core positions of CXCL10, EGF and STAT1 in the PPI network. CXCL10, a proinflammatory cytokine, has been reported to participate in the pathogenesis of BKVN^29^. The levels of CXCL10 were found to increase in serum and renal tissue of patients with BKVN as compared with those with non-rejection allografts^23,30^. We also demonstrated that CXCL10 was more expressed in patients with BKVN. All these findings indicate that CXCL10 plays a pivotal role in the immune response against BKV. According to our results, EGF appears to be another important hub protein. However, few researchers have reported the relationship between EGF and BKVN thus far. Rintala and his colleagues found that EGF played an important role in chronic allograft injury^31^. EGF interacts with TGF-β, VEGF and some other cytokines to promote tissue repair^32,33^. As per our analysis, the up-regulated CXCL10 may interact with the down-regulated EGF in the pathogenesis of BKVN. This suggests that BKV may induce tissue repair by promoting the inflammation and inhibiting the tissue repair in renal-transplantation recipients. Thus far, however, STAT1 has not been reported to associate with BKVN. Giacobbi *et al*. found that STAT1 was necessary in antiviral state and that induction of STAT1 mediated innate immune responses^34^.

We investigated the crucial proteins in BKVN through various data mining methods including the DEG analysis, GO, KEGG, literature mining, STRING and the PPI analysis. These bioinformatics methods may corroborate each other and make the result reliable. The fundamental aim of our study was to infer the potential mechanism of BKVN via bioinformatic analysis. We did not attempt to find diagnostic or prognostic biomarkers for BKVN in this sole study, because renal diseases are frequently associated with immune dysfunction. It appears difficult to identify a single renal disease only by cell factors as biomarkers. Though CXCL10 and EGF have been reported altered in a variety of renal diseases, even in the kidney rejection, the network of CXCL10, EGF and STAT1 in BKVN has not been reported. We believe that this network may provide new ideas for the elucidation of the immunological and biological mechanisms of BKVN.

Our study has some limitations. First, other non-technical site- based microarray data were not integrated in our study. Second, as Sigdel and his colleague reported, the relation between transcriptome to proteome may not be strong enough^35^. In this regard, making a protein-to-protein network from transcriptomic data might be risky. Due to the bioinformatic nature of our study, the specific mechanism and pathways of CXCL10, EGF, STAT1 and other important proteins in the PPI network were not further investigated. Therefore, animal and laboratory experiments are mandatory to further clarify the pathogenesis of BKVN. Finally, acute T cell-mediated rejection is a well-known confounder of BKVN, and we cannot rule out this confounding factor by this bioinformatic study.

In summary, we investigated the potential crucial protein network of BKVN patients. A protein network was selected by DEG, GO, KEGG and PPI analyses. CXCL10, EGF and STAT1 are hub proteins in the pathogenesis of BKVN. BKV may induce kidney injuries by promoting inflammation and prohibiting tissue reparation.

## Materials and Methods

### Affymetrix microarray data

To identify DEGs between BKVN patients and stable allograft recipients, the microarray dataset *GSE75693* were downloaded from the public Gene Expression Omnibus (GEO) database (http://www.ncbi.nlm.nih.gov/geo/). The dataset *GSE75693* was deposited by Sigdel *et al*.^35^, containing information of renal bioptic tissues from 30 stable renal allografts, 15 acute rejection patients, 15 BKVN patients and 12 chronic allograft nephropathy patients. Here, we selected the 30 stable renal allografts and the 15 BKVN patients as study subjects. The array data were based on the GPL570 Affymetrix Human Gene U133 Plus 2.0 Array (Affymetrix Inc., Santa Clara, CA, USA) sourced from renal bioptic tissues of patients. Microarray data were processed by a series of bioinformatic methods to identify the possible protein interaction network and to infer the functional process in the pathogenesis of BKVN (Supplementary Fig. S1). The raw data was preprocessed by Robust Multi-array Average^36^ algorithmin affy package of Bioconductor (http://www.bioconductor.org/), including background correction, normalization and calculation of gene expressions. For all samples in the dataset, probes for the same gene were reduced to a single value according to the maximum one^37^.

### DEGs analysis

DEGs between BKVN and non-allograft injury patients were analyzed by the *limma* package of Bioconductor. Linear models were constructed for gene expression data of BKVN and stable renal allograft samples respectively. Then the contrast model was used to compare gene expression differences between the two groups. The Student’s t-test was used to calculate the *P* values. DEGs were selected based on the threshold *P* < 0.01 and fold change >2.0. *P* value here was used to test if the gene was differentially expressed between the BKVN and the stable groups with the fold change >2.0.

### Enrichment analysis of DEGs

Gene Ontology (GO) and the Kyoto Encyclopedia of Genes and Genomes (KEGG) are two aspects of DEGs enrichment analysis, which helps us to learn the potential mechanism of BKVN. GO annotated genes by a defined, structured and controlled vocabulary^38^, including molecular function (MF), biological process (BP) and cellular components (CC), while KEGG assigns DEGs to specific pathways^39^. GO and KEGG can be performed by Database for Annotation, Visualization and Integrated Discovery (DAVID, http://david.abcc.ncifcrf.gov/). We analyzed biological functions behind massive genes with *P* < 0.05 and the count ≥5.

### PPI network construction and literature mining

PPI shows the potential network and connections of *DEGs*. PPI is usually done by STRING (Search Tool for the Retrieval of Interacting Genes/Proteins, http://string-db.org/), which is a web source of biological database. List of DEGs was uploaded onto the STRING. According to the official explanation of STRING, the confidence score is the approximate probability that a predicted link exists between two proteins in the same metabolic map in the KEGG database (https://string-db.org/cgi/help.pl?UserId=rtxtBR80pDyg&sessionId=si2cM9wdJB3P). Thus, PPIs of DEGs were selected with the threshold of score (median confidence) >0.4^36,39^. Then the analysis results of PPIs were downloaded from STRING, and modified by Cytoscape (http://www.cytoscape.org/). According to the analysis of STRING, nodes with higher degree in the PPI were put into GenCLiP 2.0 (http://ci.smu.edu.cn/GenCLiP2.0/confirm_keywords.php), which is an online tool for literature mining of genes^40^. GenCLiP could generate keywords of genes in previous literatures to help us infer the possible gene function^40^. In GenCLiP, biological keywords of hot genes in previous studies were analyzed by the “Gene Cluster with Literature Profiles” module with *P* ≤ 1 × 10^−4^ and hit ≥4. And the co-citation network of hot genes was selected by “Literature Mining Gene Networks” module.

### Hub protein selection by CytoNCA

In Cytoscape, scattered proteins were removed from the final PPIs. The hub protein, which interacts most frequently with other proteins and works like a hub in the network, were selected by CytoNCA according to degree centrality, betweenness centrality and subgraph centrality^36^. Finally, proteins associated with hub proteins at the degree ≥10 were selected and constructed the significant network of BKVN mechanism^39^.

### Data availability statement

The *GSE75693* dataset analyzed during the current study is available in the Gene Expression Omnibus database (http://www.ncbi.nlm.nih.gov/geo/).

## Electronic supplementary material

Supplementary Files

Supplementary Dataset 1–5

## References

[1] Ambalathingal GR, Francis RS, Smyth MJ, Smith C, Khanna R (2017). BK Polyomavirus: Clinical Aspects, Immune Regulation, and Emerging Therapies. Clin Microbiol Rev.

[2] Knowles, W. A. In Human Polyomaviruses: Molecular and Clinical Perspectives. (eds K. Khalili & G. L. Stoner) 527–559 (Wiley-Liss Inc, New York; 2001).

[3] Hirsch HH, Steiger J (2003). Polyomavirus BK. Lancet Infect Dis.

[4] Drachenberg CB (2004). Histological patterns of polyomavirus nephropathy: correlation with graft outcome and viral load. Am J Transplant.

[5] Hirsch HH, Randhawa P (2009). BK virus in solid organ transplant recipients. Am J Transplant.

[6] Jaw J, Hill P, Goodman D (2017). Combination of Leflunomide and Everolimus for treatment of BK virus nephropathy. Nephrology (Carlton).

[7] Alagoz S (2017). The Frequency and Associated Factors for BK Virus Infection in a Center Performing Mainly Living Kidney Transplantations. Prog Transplant.

[8] Brennan DC (2005). Incidence of BK with tacrolimus versus cyclosporine and impact of preemptive immunosuppression reduction. Am J Transplant.

[9] Hirsch HH (2013). Polyomavirus BK replication in de novo kidney transplant patients receiving tacrolimus or cyclosporine: a prospective, randomized, multicenter study. Am J Transplant.

[10] Shen CL, Yang AH, Lien TJ, Tarng DC, Yang CY (2017). Tacrolimus Blood Level Fluctuation Predisposes to Coexisting BK Virus Nephropathy and Acute Allograft Rejection. Sci Rep.

[11] Kable K, Davies CD, O’Connell P J, Chapman JR, Nankivell BJ (2017). Clearance of BK Virus Nephropathy by Combination Antiviral Therapy With Intravenous Immunoglobulin. Transplant Direct.

[12] Drake DR (2000). Polyomavirus-infected dendritic cells induce antiviral CD8(+) T lymphocytes. J Virol.

[13] Drake DR (2001). Induction of polyomavirus-specific CD8(+) T lymphocytes by distinct dendritic cell subpopulations. J Virol.

[14] Bauman Y (2011). An identical miRNA of the human JC and BK polyoma viruses targets the stress-induced ligand ULBP3 to escape immune elimination. Cell Host Microbe.

[15] Ribeiro A (2012). Activation of innate immune defense mechanisms contributes to polyomavirus BK-associated nephropathy. Kidney Int.

[16] Kimball AB, Grant RA, Wang F, Osborne R, Tiesman JP (2012). Beyond the blot: cutting edge tools for genomics, proteomics and metabolomics analyses and previous successes. Br J Dermatol.

[17] Foulkes AC (2017). Research Techniques Made Simple: Bioinformatics for Genome-Scale Biology. J Invest Dermatol.

[18] Mele M (2015). Human genomics. The human transcriptome across tissues and individuals. Science.

[19] Mou T (2017). Identification and interaction analysis of key genes and microRNAs in hepatocellular carcinoma by bioinformatics analysis. World J Surg Oncol.

[20] Yoshioka Y (2017). Protein lysine methyltransferase SMYD3 is involved in tumorigenesis through regulation of HER2 homodimerization. Cancer Med.

[21] Jung K (2015). Gene expression profile of necrotizing enterocolitis model in neonatal mice. Int J Surg.

[22] Gatto M (2013). Serpins, immunity and autoimmunity: old molecules, new functions. Clin Rev Allergy Immunol.

[23] Kariminik A, Dabiri S, Yaghobi R (2016). Polyomavirus BK Induces Inflammation via Up-regulation of CXCL10 at Translation Levels in Renal Transplant Patients with Nephropathy. Inflammation.

[24] Hickman HD (2015). CXCR3 chemokine receptor enables local CD8(+) T cell migration for the destruction of virus-infected cells. Immunity.

[25] Panzer U (2004). CXCR3 and CCR5 positive T-cell recruitment in acute human renal allograft rejection. Transplantation.

[26] Hodge DL (2002). IL-2 and IL-12 alter NK cell responsiveness to IFN-gamma-inducible protein 10 by down-regulating CXCR3 expression. J Immunol.

[27] Schorey JS, Cheng Y, Singh PP, Smith VL (2015). Exosomes and other extracellular vesicles in host-pathogen interactions. EMBO Rep.

[28] Dreux M (2012). Short-range exosomal transfer of viral RNA from infected cells to plasmacytoid dendritic cells triggers innate immunity. Cell Host Microbe.

[29] Tatapudi RR (2004). Noninvasive detection of renal allograft inflammation by measurements of mRNA for IP-10 and CXCR3 in urine. Kidney Int.

[30] Hu H (2004). Elevation of CXCR3-binding chemokines in urine indicates acute renal-allograft dysfunction. Am J Transplant.

[31] Rintala JM (2014). Epidermal growth factor inhibition, a novel pathway to prevent chronic allograft injury. Transplantation.

[32] Petit AM (1997). Neutralizing antibodies against epidermal growth factor and ErbB-2/neu receptor tyrosine kinases down-regulate vascular endothelial growth factor production by tumor cells *in vitro* and *in vivo*: angiogenic implications for signal transduction therapy of solid tumors. Am J Pathol.

[33] Cappuzzo F (2010). Erlotinib as maintenance treatment in advanced non-small-cell lung cancer: a multicentre, randomised, placebo-controlled phase 3 study. Lancet Oncol.

[34] Giacobbi NS, Gupta T, Coxon AT, Pipas JM (2015). Polyomavirus T antigens activate an antiviral state. Virology.

[35] Sigdel TK (2016). Mining the human urine proteome for monitoring renal transplant injury. Kidney Int.

[36] Lin Z, Lin Y (2017). Identification of potential crucial genes associated with steroid-induced necrosis of femoral head based on gene expression profile. Gene.

[37] Subramanian A (2005). Gene set enrichment analysis: a knowledge-based approach for interpreting genome-wide expression profiles. Proc Natl Acad Sci USA.

[38] Ashburner M (2000). Gene ontology: tool for the unification of biology. The Gene Ontology Consortium. Nat Genet.

[39] Li L (2017). Identification of key genes and pathways associated with obesity in children. Exp Ther Med.

[40] Wang JH (2014). GenCLiP 2.0: a web server for functional clustering of genes and construction of molecular networks based on free terms. Bioinformatics.

