# Supplementary material for: Identification of potential key protein interaction networks of BK virus nephropathy in patients receiving kidney transplantation
Source: Sci Rep. 2018 Mar 22;8:5017. doi: 10.1038/s41598-018-23492-2 (PMC5864740; doi:10.1038/s41598-018-23492-2)
Supplement: Supplementary file 1 — Supplementary Files [file 41598_2018_23492_MOESM1_ESM.pdf]

Identification of potential key protein interaction networks of BK virus nephropathy in patients receiving kidney transplantation

Linpei Jia[1]*, Wenjing Fu[1], Rufu Jia[2], Leiyun Wu[1], Xiaoxia Li[1], Qiang Jia[1]*, Hongliang Zhang[3]*

[1]Department of Nephrology, Xuanwu Hospital of Capital Medical University, Changchun Street 45#, 100053, Beijing, China.

[2]Central Hospital of Cangzhou, Xinhua Middle Street 201#, 061001, Cangzhou, Hebei Province, China.

[3]Department of Life Sciences, the National Natural Science Foundation of China, Shuangqing Road 83#, 100085, Beijing, China.

***Address correspondence to:**

Dr. Linpei Jia, Department of Nephrology, Xuanwu Hospital of Capital Medical University, Changchun Street 45#, 100053, Beijing, China, Email: anny_069@163.com

Dr. Hongliang Zhang, Department of Life Sciences, the National Natural Science Foundation of China, Shuangqing Road 83#, 100085, Beijing, China, Email: drzhl@hotmail.com.

Dr. Qiang Jia, Department of Nephrology, Xuanwu Hospital of Capital Medical University, Changchun Street 45#, 100053, Beijing, China. Email: jiaqiang9509@sina.com.

Linpei Jia, Hongliang Zhang and Qiang Jia contributed equally to the work as the correspondence authors.

Supplementary Table S1 Immune mediators possibly involved in the pathogenesis of BK virus nephropathy.

Type	Immune mediators
Cytokines	IL-2, IL-6, IL-8, MCP-1/CCL2[1], TGF-β[2], IFN-γ and TNF[3].
Chemokines	CXCL8, RANTES, CCL5, MCP-1, CCL2, IP-10 and CXCL10[1].
Transcription factor	STAT1[4].
Others	MMP2 and MMP9[2].

Reference

1. Ribeiro, A. et al. Activation of innate immune defense mechanisms contributes to polyomavirus BK-associated nephropathy. *Kidney Int* **81**, 100-111 (2012).
2. Mannon, R.B. et al. Molecular evaluation of BK polyomavirus nephropathy. *Am J Transplant* **5**, 2883-2893 (2005).
3. Weist, B.J. et al. The role of CD4(+) T cells in BKV-specific T cell immunity. *Med Microbiol Immunol* **203**, 395-408 (2014).
4. Giacobbi, N.S., Gupta, T., Coxon, A.T. & Pipas, J.M. Polyomavirus T antigens activate an antiviral state. *Virology* **476**, 377-385 (2015).

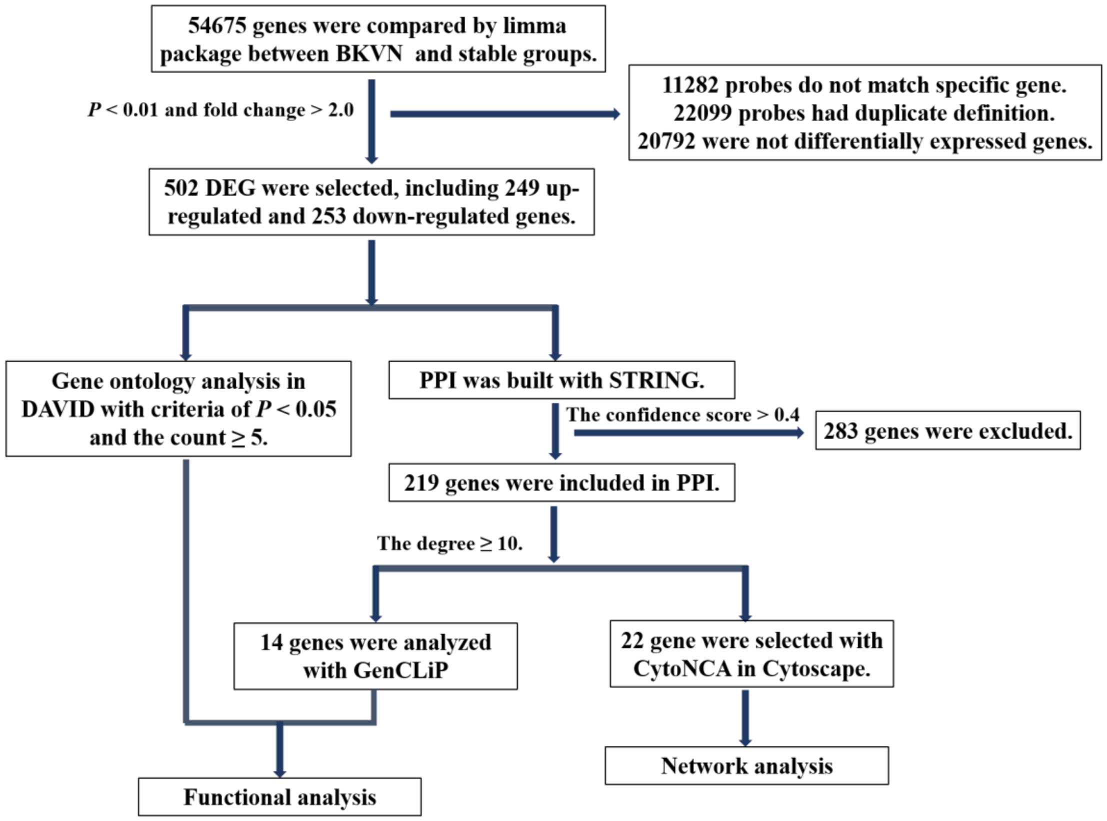

Supplementary Figure S1 Flow chart of key network selection in BK virus nephropathy (BKVN). In the microarray data, 54675 were compared between the BKVN and the stable kidney transplantation patients. After excluding duplicate and non-specific genes, 502 differentially expressed genes (DEGs) were selected by limma package. Then protein-protein interactions (PPIs) were analyzed by STRING website, and 219 DEGs were included. PPI were further estimated by CytoNCA in Cytoscape software. A protein interacting network was finally selected, which included 22 important proteins in BKVN pathogenesis. Meanwhile, we put 502 DEGs into DAVID website to find out the potential biological process and pathways involved in BKVN. In the PPI network, we further analyzed the biological functions of 14 important significant proteins by GenCLiP software.
